# Covid‐19 dermatoses: Acral vesicular pattern evolving into bullous pemphigoid

**DOI:** 10.1002/ski2.6

**Published:** 2020-11-11

**Authors:** P.K.C. Goon, O. Bello, L.A. Adamczyk, J.Y.H. Chan, H. Sudhoff, C.C. Banfield

**Affiliations:** ^1^ Department of Dermatology Peterborough City Hospital North West Anglia Foundation Trust (NWAFT) Peterborough UK; ^2^ Department of Histopathology Peterborough City Hospital North West Anglia Foundation Trust (NWAFT) Peterborough UK; ^3^ Addenbrooke's Hospital Cambridge University Hospitals NHS Foundation Trust Cambridge UK; ^4^ University Hospital of Bielefeld Bielefeld Germany

## Abstract

Bullous pemphigoid (BP) appears to be rising in incidence across the Western World, especially in the elderly. Some of the pathogenetic mechanisms involving antigen mimicry and antibody cross‐reactivity have been elucidated for cases associated with neurological disease and certain drugs. There have been reports of cutaneous manifestations of Covid‐19 (SARS‐Cov2 infection) as the pandemic has raged across the world. We report here a case of prolonged Covid‐19, symptomatic with dermatoses only, which was seen to evolve initially from a maculo‐papular exanthema with acral vesicular dermatitis, into classical BP disease. This was confirmed histologically by positive skin autoantibody serology, direct IMF on peri‐lesional skin and also salt‐split IMF. Although possible that the development of BP could be a purely co‐incidental finding during Covid‐19, we suggest that it is more likely that prolonged SARS‐Cov2 infection triggered an autoimmune response to the basement membrane antigens, BP 180 and 230. To our knowledge, this is the first case of BP developing during concurrent Covid‐19 disease. It will be necessary to continue dermatological surveillance as the pandemic continues, to collate data on BP incidence and to test these patients for Covid‐19 disease. As the pandemic continues, even potential and rare associations such as this will be clarified eventually.

What's already known about this topic?

- Covid‐19 disease has been associated with a spectrum of dermatoses
- Common presentations in up to 20% of patients include exanthema, pseudo‐chilblain like acral lesions ‘Covid toes’, livedo‐/retiform purpuric/necrotic vascular lesions, acute urticarial lesions, and vesicular/varicella‐like lesions
- A multi‐system inflammatory syndrome in children akin to Kawasaki syndrome has been described

What does this study add?

- To our knowledge, this is the first description of classic Bullous Pemphigoid evolving from vesicular lesions caused by prolonged SARS‐Cov2 induced skin inflammation

## 1 CASE REPORT

Bullous pemphigoid (BP) is a rare disease (<5/100,000)^1^ but reported to be increasing in incidence, particularly in the over 70s.^2^, ^3^, ^4^ The various risk factors associated with this rise are the increasingly elderly populations in Western Europe, an increasing use of drugs such as dipeptidyl‐peptidase IV inhibitors (DPP4i) in the treatment of type 2 diabetes mellitus,^5^, ^6^ psychotropic drugs (particularly phenothiazines with aliphatic side chains),^7^ checkpoint inhibitors such as anti‐PD‐1, and PD‐L1^8^ and neurological disease burden such as dementia, stroke, multiple sclerosis, and so on^9^, ^10^ amongst the elderly. Cross reactivity between the neuronal and epithelial isoforms of BP230^9^ ^,^ ^10^, inhibition of plasmin by DPP4i altering cleavage of BP180 within the NC‐16A domain^11^ and other not yet elucidated mechanisms are thought to be responsible for the rising incidence of BP.

The American Academy of Dermatology and the British Association of Dermatologists have categorised several of the most common, mainly referenced by studies from Spain^12^ and Italy.^13^, ^14^ None report an association with autoimmune bullous disease. Here we present a case of BP arising during prolonged Covid‐19 disease for 6 weeks.

An 82‐year‐old female was admitted to hospital at the end of April after an episode of angina pectoris. Echocardiography revealed critical aortic stenosis with prognosis expected to be less than 1 year if untreated. Her past medical history included ischaemic heart disease, TIA (transient ischaemic attack), hypertension and hyperlipidaemia. Her medications on admission were clopidogrel, aspirin, atenolol, furosemide, omeprazole, Vit D3, amlodipine, atorvastatin and ramipril. She was then worked up for transfer to the regional tertiary referral centre for cardiothoracic medicine for Transcatheter Aortic Valve implantation (TAVI). As part of her work‐up, she tested positive for SARS‐Cov2 by RT‐PCR, and continued to test positive weekly for 6 weeks in a row. Her procedure and transfer had to be deferred (until 2 negative results were obtained a week apart). She reported no respiratory symptoms, did not require O_2_ as her O_2_ saturation percentages were excellent at rest, and was admitted to an isolation room on the cardiac ward.

**FIGURE 1:**
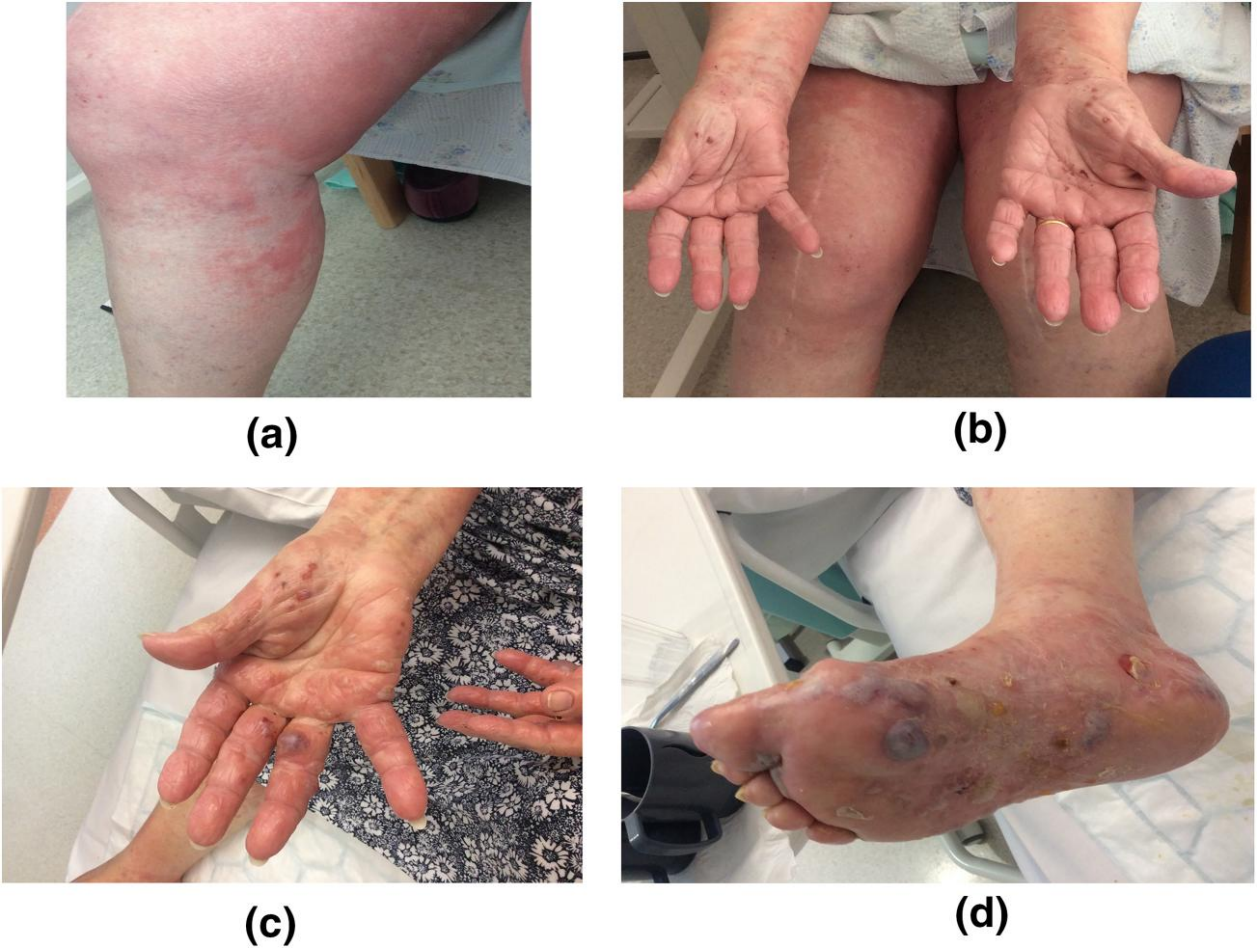
(a) Maculo‐papular exanthem. (b) Acro‐vesicular dermatitis. (c) Bullae on the hands. (d) Bullae on the feet

In week 3, she developed a skin rash which was itchy and started initially on the trunk, and gradually spread onto her limbs. In week 4, the decision was taken to ask for a dermatology consultation as the rash was progressing and not responding to treatment. The rash was diagnosed as a viral exanthem, with a fairly classic maculo‐papular rash (see Figure 1), associated with Covid 19. The patient had also developed small acral vesicular lesions which were excoriated (see Figure 1). These patterns have been described in Covid19.^12^ The decision was taken to start the patient on super potent topical steroids in addition to greasy emollients.

**FIGURE 2:**
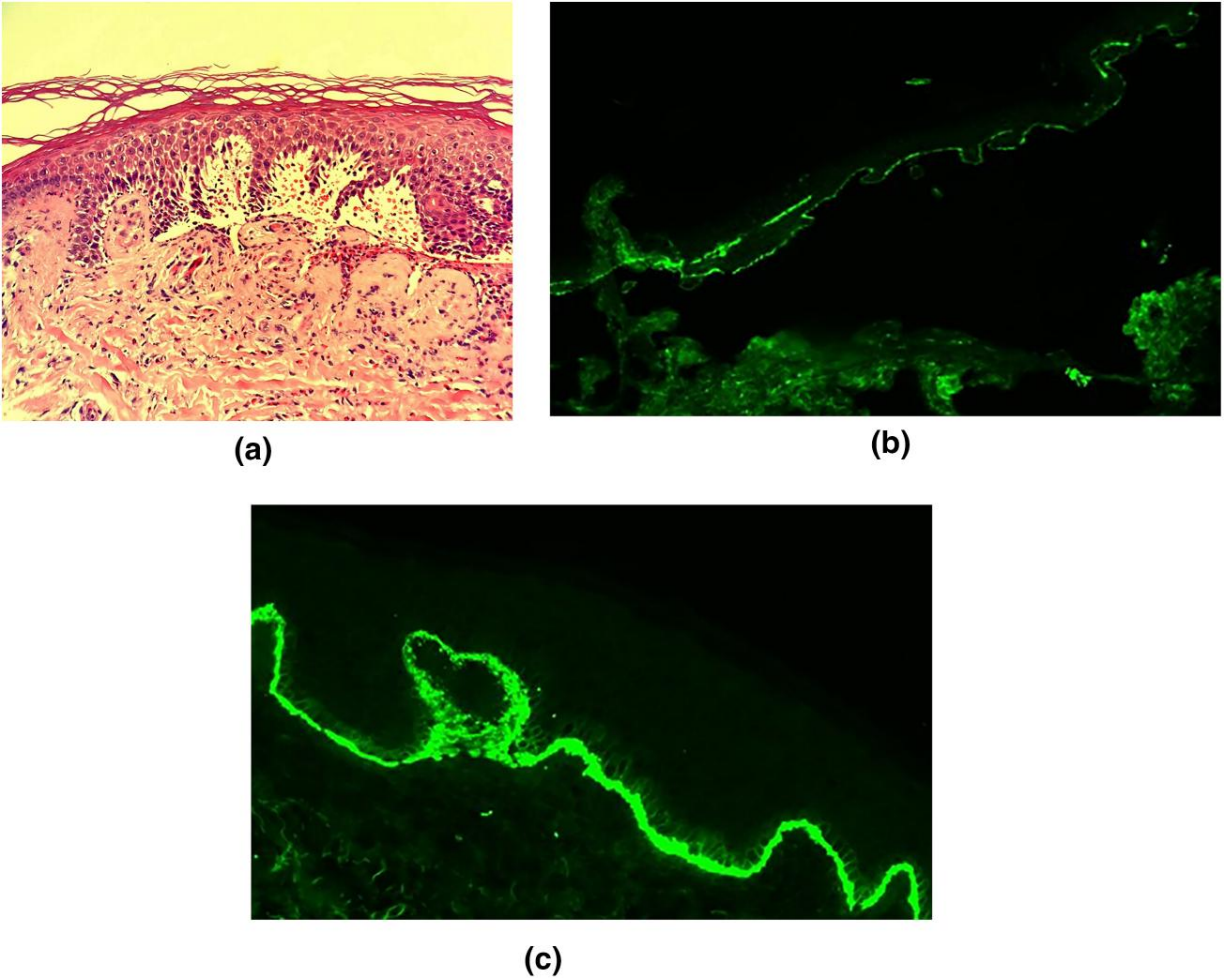
(a) Haematoxylin and Eosin staining of a biopsy from edge of a blister. Histopathological examination revealed typical features of BP with a subepidermal blister filled with fibrin, neutrophils and eosinophils and festooning of the epidermal papillae. (b) ‘Gold standard’ confirmation of clinically diagnosed Bullous Pemphigoid by Direct Immunofluorescence staining. IgG‐salt split (staining strongly positive for IgG on the roof of the blister). (c) C3 staining

In week 5, Dermatology was again asked to review the patient, as the patient was now complaining of soreness and discomfort on her hands, and the cardiology team now reported that larger blisters had developed. The truncal and limb rash had now essentially resolved. Further examination now revealed that the patient had developed tense bullae, typical of BP (see Figure 1). Blood testing for skin autoantibodies (indirect IMF) and diagnostic biopsies were taken for H&E and Direct IMF staining. Results are shown in Figure 2 [NB: The definitions of vesicles are blisters less than 5 mm and bullae are blisters over 5 mm].

The patient was started on a reducing regimen of oral Prednisolone, plus oral Doxycycline. She responded well to this new regimen, and there were no new bullae after a few days with near complete resolution on her hands, and healing bullae on her feet after a week. She subsequently tested negative for SARS‐Cov2 in weeks 7 and 8 and was transferred to the regional Cardiothoracic hospital for her TAVI, which has now been successfully performed. Dermatology review 4 weeks after she got home for recuperation confirmed her continued skin recovery.

## 2 DISCUSSION

We hypothesise that prolonged inflammation of the skin during the initial phase of the viral exanthem has left the damaged basement membrane open and susceptible to immune recognition by the host's immune system, and the subsequent development of autoantibodies to BP antigens 180 or 230, possibly through antigenic mimicry of viral antigens in an analogous manner to ‘Fogo Selvagem’. This is an endemic autoimmune bullous disease seen in clusters along rivers and waterways in Brazil and South America. It has been hypothesized that bites by *Simulium nigrimanum* triggers an antibody response to EC‐5 (an antigenic domain of Desmoglein‐1, responsible for the pre‐clinical phase), and in susceptible individuals, subsequent responses to EC1‐2 domains develop by epitope spreading, and thus full‐blown ‘Wildfire’ of the skin.^15^

Are viruses or viral vaccines potential triggers for autoimmune bullous disease?

Sagi et al.^16^ have shown that in their cohort, patients with autoimmune bullous disease were found to have significantly higher prevalence of IgG antibodies *demonstrating past infection* with viruses such as HBV, HCV and CMV compared to controls but the authors did not suggest that this implied causality. There have also been reports of flu vaccines (including swine flu) triggering BP and again the mechanisms suggested have been skin inflammation and exposure of basement membrane proteins to the immune response.^17^ ^,^ ^18^

We have described a case of a patient who developed Covid19 maculo‐papular and acral vesicular rashes initially, and this then evolved into BP during prolonged SARS‐Cov2 infection. The estimated incidence rate of Covid19 infection in the over 70s in the United Kingdom in this period, was 0.3% or 3 per 1000 (a) Office of National Statistics (Accessed 24 Sep, 2020) https://www.ons.gov.uk/peoplepopulationandcommunity/healthandsocialcare/conditionsanddiseases/bulletins/coronaviruscovid19infectionsurveypilot/england21may2020. The incidence rate of BP is less than 5 per 100,000 (b). Therefore, the chances of a completely unrelated but concurrent manifestation of both diseases was less than 0.15 per 1 million (a x b).

It is therefore more plausible to assume that her skin disease was indeed associated with her viral infection, rather than random and purely coincidental. This rare association has not been reported so far in the pandemic, and it will be useful to test BP patients for SARS‐Cov2 infection during this pandemic to determine whether this is a true but rare association.

## CONFLICT OF INTERESTS

No conflict of interests have been declared.

## References

[1] Kridin K , Ludwig RJ . The growing incidence of bullous pemphigoid: overview and potential explanations. Front Med. 2018;5:220. 10.3389/fmed.2018.00220 PMC6109638 30177969

[2] Hubner F , Recke A , Zillikens D , Linder R , Schmidt E . Prevalence and age distribution of pemphigus and pemphigoid diseases in Germany. J Invest Dermatol. 2016;136:2495–2498. 27456755 10.1016/j.jid.2016.07.013

[3] Joly P , Baricault S , Sparsa A , et al. Incidence and mortality of bullous pemphigoid in France. J Invest Dermatol. 2012;132:1998–2004. 22418872 10.1038/jid.2012.35

[4] Langan SM , Smeeth L , Hubbard R , et al. Bullous pemphigoid and pemphigus vulgaris–incidence and mortality in the UK: population based cohort study. BMJ. 2008;337:a180. 18614511 10.1136/bmj.a180 PMC2483869

[5] Benzaquen M , Borradori L , Berbis P , et al. Dipeptidyl peptidase IV inhibitors, a risk factor for bullous pemphigoid: Retrospective multicenter case‐control study from France and Switzerland. J Am Acad Dermatol. 2018;78:1090–6. 29274348 10.1016/j.jaad.2017.12.038

[6] Varpuluoma O , Forsti AK , Jokelainen J , et al. Vildagliptin significantly increases the risk of bullous pemphigoid: a Finnish nationwide Registry study. J Invest Dermatol. 2018;138:1659–61. 29427585 10.1016/j.jid.2018.01.027

[7] Bastuji‐Garin S , Joly P , Lemordant P , et al. Risk factors for bullous pemphigoid in the elderly: a prospective case‐control study. J Invest Dermatol. 2011;131:637–43. 20944650 10.1038/jid.2010.301

[8] Lopez AT , Khanna T , Antonov N , et al. A review of bullous pemphigoid associated with PD‐1 and PD‐L1 inhibitors. Int J Dermatol. 2018;57:664–9. 29630716 10.1111/ijd.13984

[9] Brown A , Bernier G , Mathieu M , et al. The mouse dystonia musculorum gene is a neural isoform of bullous pemphigoid antigen 1. Nat Genet. 1995;10:301–6. 7670468 10.1038/ng0795-301

[10] Seppanen A , Autio‐Harmainen H , Alafuzoff I , et al. Collagen XVII is expressed in human CNS neurons. Matrix Biol. 2006;25:185–8. 16387484 10.1016/j.matbio.2005.11.004

[11] Izumi K , Nishie W , Mai Y , et al. Autoantibody profile differentiates between inflammatory and noninflammatory bullous pemphigoid. J Invest Dermatol. 2016;136:2201–10. 27424319 10.1016/j.jid.2016.06.622

[12] Galvan Casas C , Catala A , Carretero Hernandez G , et al. Classification of the cutaneous manifestations of COVID‐19: a rapid prospective nationwide consensus study in Spain with 375 cases. Br J Dermatol. 2020;183:71–7. 32348545 10.1111/bjd.19163 PMC7267236

[13] Gianotti R , Recalcati S , Fantini F , et al. Histopathological study of a broad spectrum of skin dermatoses in patients affected or highly suspected of infection by COVID‐19 in the northern part of Italy: analysis of the many faces of the viral‐induced skin diseases in previous and new reported cases. Am J Dermatopathol. 2020;42(8):564–570. 32701690 10.1097/DAD.0000000000001707 PMC7368844

[14] Recalcati S . Cutaneous manifestations in COVID‐19: a first perspective. J Eur Acad Dermatol Venereol. 2020;34:e212–e3. 32215952 10.1111/jdv.16387

[15] Rodrigues DB , Pereira SA , dos Reis MA , et al. In situ detection of inflammatory cytokines and apoptosis in pemphigus foliaceus patients. Arch Pathol Lab Med. 2009;133:97–100. 19123745 10.5858/133.1.97

[16] Sagi L , Baum S , Agmon‐Levin N , et al. Autoimmune bullous diseases the spectrum of infectious agent antibodies and review of the literature. Autoimmun Rev. 2011;10:527–35. 21527361 10.1016/j.autrev.2011.04.003

[17] Downs AM , Lear JT , Bower CP , et al. Does influenza vaccination induce bullous pemphigoid? A report of four cases. Br J Dermatol. 1998;138:363. 10.1046/j.1365-2133.1998.02097.x 9602897

[18] Walmsley N , Hampton P . Bullous pemphigoid triggered by swine flu vaccination: case report and review of vaccine triggered pemphigoid. J Dermatol Case Rep. 2011;5:74–6. 22408707 10.3315/jdcr.2011.1081 PMC3241950

